# Using the Rise and Fall of Oxidative Stress and Inflammation Post-Exercise to Evaluate the Effect of Methylsulfonylmethane Supplementation on Immune Response mRNA

**DOI:** 10.3390/nu17111761

**Published:** 2025-05-23

**Authors:** Brian K. McFarlin, John H. Curtis, Heidi N. du Preez, Meredith A. McFarlin

**Affiliations:** 1 Applied Physiology Laboratory, University of North Texas, Denton, TX 76205, USA; john.curtis@unt.edu (J.H.C.); meredith.mcfarlin@unt.edu (M.A.M.); 2 Bioanalysis Center, University of North Texas, Denton, TX 76205, USA; 3 Catalysis and Peptide Research Unit, University of KwaZulu-Natal, Durban 4041, South Africa; hdp@heididupreez.com

**Keywords:** muscle fatigue, muscle injury, immune cell activation, MSM

## Abstract

**Background:** Long-duration aerobic exercise results in a similar, albeit transient rise and fall in oxidative stress and inflammation, making it a useful model to evaluate nutritional supplements targeting these physiological processes. **Objective:** To evaluate the impact of MSM supplementation on post-exercise immune response-related mRNA expression. **Methods:** In the present study, we enrolled healthy, experienced runners (five MSM and five placebo) who were supplemented with Methylsulfonylmethane (MSM; 1.0 g/d) or placebo for 30 days prior to a 21.1 km running event (120 to 150 min). Venous blood samples were collected prior to (PRE) the event, as well as 2 h and 4 h after the event to measure the expression of 700 mRNAs associated with generalized immune response. **Results:** This study is the first to demonstrate significant effects with lower MSM doses (0.5–1.0 g/d) compared to previous work using higher doses (3 g/d). We identified 29 mRNAs in four distinct immune response pathways (peripheral tissue inflammatory response, myeloid immune cell invasion, NK cell invasion/activity, and notch signaling) whose response was statistically changed with MSM at 2 h and/or 4 h. **Conclusions:** Based on the physiologic actions of the mRNA that changed, some logical potential health effects of MSM may be that it helps with the following: (1) supports muscle recovery by improving macrophage response to exercise, (2) speeds up recovery and restoration of damaged muscle tissue, (3) supports innate immune responsiveness to DAMP, and (4) reduces and/or improves resistance to oxidative stress after exercise. Future research should seek to validate how the changes observed with exercise may model to various chronic inflammatory states.

## 1. Introduction

Atherosclerosis, rheumatoid arthritis, and related diseases are caused by chronic elevations in oxidative stress and inflammation [1,2]. These disease states are challenging to study due to the presence of various confounding factors, individual differences in disease progression and genetic predisposition, and the ethics of working with diseased individuals [1]. To address these limitations, one approach may be to use a physiological stressor that results in a similar, acute rise and fall of oxidative stress and inflammation when evaluating compounds with the potential to alter these outcomes. One such stimulus is long-duration aerobic exercise, which has minimal long-term adverse effects in healthy individuals [3,4]. While there are many existing pharmaceuticals available to address chronic disease, the identification of novel, prophylactic, preventative nutritional treatments may be warranted. Methylsulfonylmethane (MSM) has been reported as an effective nutraceutical that reduces the symptoms in various chronic inflammatory disease [5,6,7,8]. Mechanistically, MSM has antioxidant, anti-inflammatory, immune boosting, and active sulfur/methyl donor effects [9,10,11,12,13]. When consumed orally for short periods (<21 days), MSM absorbs rapidly and has a high degree of bioavailability in humans with no known adverse effects [9,13].

Touguchi et al. reported a reduction in knee osteoarthritis symptoms after 12 weeks of MSM (2 g/d) supplementation [7]. A systematic review also indicated that MSM supplementation (1–3 g/d) was an effective treatment for arthritis-associated symptoms [8]. Using a half marathon exercise model, Withee et al. reported that MSM supplementation (3 g/d) was associated with reduced post-race subjective muscle and joint soreness [14]. We previously reported that MSM combined with other polyphenols improved post-half marathon inflammatory response [3,15] and improved immune response to lipopolysaccharide (LPS) after downhill running [16]. We designed and conducted the present study to investigate the actions of MSM alone using a half marathon model. Given our previous findings, we exclusively used mRNA biomarkers as they are sensitive to subtle changes in immune homeostasis. The purpose of the present study was to evaluate the efficacy of MSM supplementation on post-exercise changes in mRNA biomarkers, associated with immune responsiveness to oxidative stress and sterile inflammation.

## 2. Materials and Methods

### 2.1. Participants and MSM Supplementation

The Institutional Review Board (IRB) at the University of North Texas approved all aspects of the present study that was conducted in accordance with the Declaration of Helsinki (IRB # IRB-18-016; approved on 15 May 2018). All participants gave informed consent prior to participating in the study. Participants were excluded if they reported diagnosed chronic disease, regular MSM supplementation, habitual intake of pain relievers (i.e., ibuprofen, acetaminophen, aspirin), and/or contraindications to exercise. Experienced runners (38 ± 6 y; BMI 24.2 ± 2.9; running history 6 ± 4 y) who were planning to run a half marathon in the next 30 d were randomized to either MSM (*n* = 5, 2 female) or placebo (*n* = 5, 2 female) using double blind procedures. This sample size was selected based on our previous studies of a similar size using Nanostring outcome measures [3,15,16]. We used a progressive supplementation schedule consistent with our previous studies [3,15]. Briefly, for the first 27 days, participants consumed 0.5 g/d of MSM (OptiMSM^®^; Balchem; Vancouver, WA, USA) in capsule form. The following three days, the daily MSM dose was increased to 1.0 g/d. The half marathon (21.1 km) took place on the 30th day of supplementation. Previous research has studied exercise health claims at 3 g/d [14]; to our knowledge, the present study is the first to use MSM supplementation at 0.5 to 1.0 g/d.

### 2.2. Blood Collection, RNA Isolation, and Analysis

Collection and subsequent mRNA expression analysis was completed using procedures we have described in detail elsewhere [15,16,17,18,19]. In this study, venous stabilized blood samples (PreAnalytiX, Hombrechtikon, Switzerland) were collected 24 h before (PRE) and two (2 h) and four hours (4 h) after the half marathon performance. The timing of sample collection was based on results from previous studies [15]. The PRE sample was collected 24 h prior to the half marathon to minimize the disturbance of the participant’s normal schedule on race morning. Isolated total RNA was analyzed for the expression of 785 mRNAs associated with 47 immune response pathways (Human Immune Exhaustion Panel; Sprint nCounter; Nanostring, Seattle, WA, USA).

### 2.3. Statistical Methods

Our laboratory has validated a statistical method approach for using mRNA biomarkers to evaluate the efficacy of nutraceuticals such as MSM [17,20,21,22]. All normalization and statistical analyses were completed using nSolver Advanced Analysis (Nanostring). The challenge of working with many dependent variables lies with separating true from false positives. As an initial control for false discovery rate (FDR), we used Benjamini–Hochberg adjusted *p*-values to correct for multiple outcomes and comparisons. Data from MSM and placebo were analyzed separately, and differential mRNA expression was calculated by setting the PRE expression for a given mRNA as the zero point. This resulted in four differential expression points per mRNA (MSM-PRE vs. 2 h, MSM-PRE vs. 4 h, placebo-PRE vs. 2 h, placebo-PRE vs. 4 h). When a given mRNA expression changed in the same direction (either upregulated or downregulated) for both MSM and placebo it was eliminated from the dataset as it was not uniquely associated with MSM, but rather the generalized response to running a half marathon. To identify biological pathways associated with the significant mRNA, we used a series of keyword searches with the Elsevier Scopus AI tool (https://www.elsevier.com/products/scopus/scopus-ai; URL accessed on 1 January 2025 to 1 March 2025). This tool only reviewed the peer-reviewed literature and provided a useful tool for summarizing related research. This tool was not used to generate any of the written material in this manuscript.

## 3. Results

**Figure 1:**
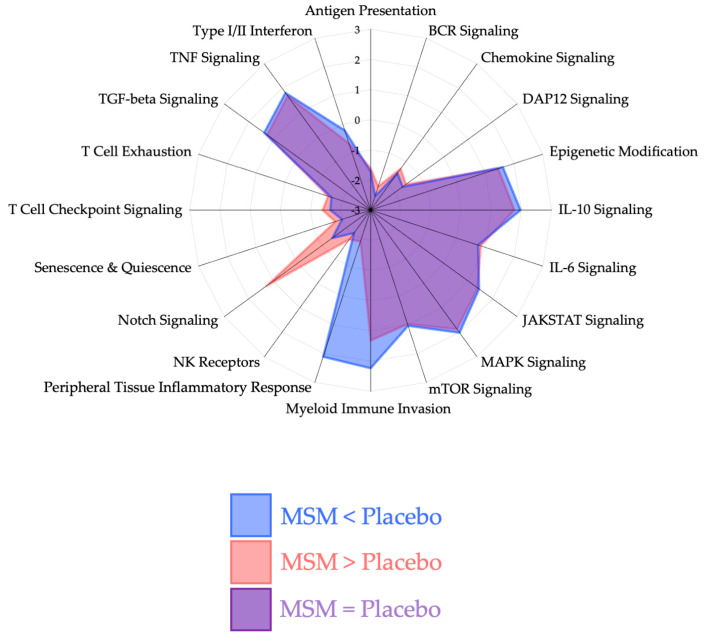
Pathway changes between MSM and placebo at 2 h after running a half marathon. MSM was associated with a differential response in the following pathways: peripheral tissue inflammatory response, myeloid immune invasion, and notch signaling.

**Figure 2:**
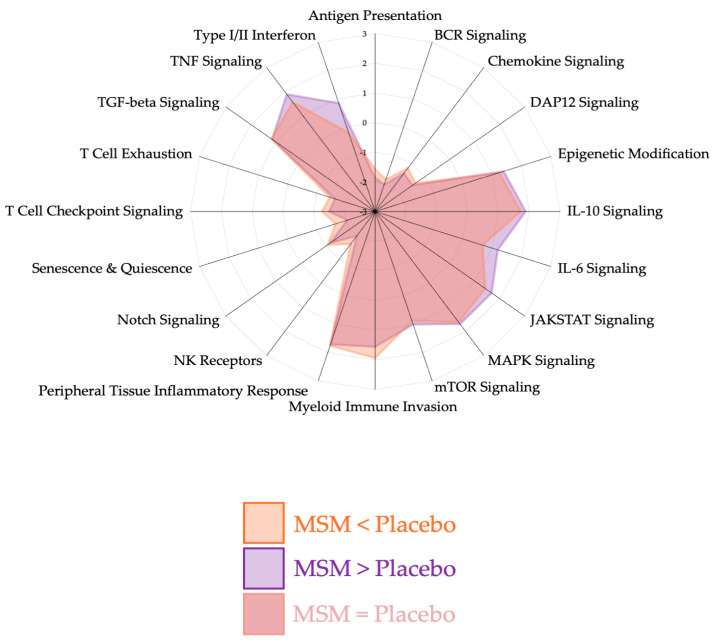
Pathway changes between MSM and placebo at 4 h after running a half marathon. MSM was associated with a differential response in the following pathways: peripheral tissue inflammatory response, myeloid immune invasion, and notch signaling. These changes were less pronounced than what was observed at 2 h post-exercise.

**Figure 3:**
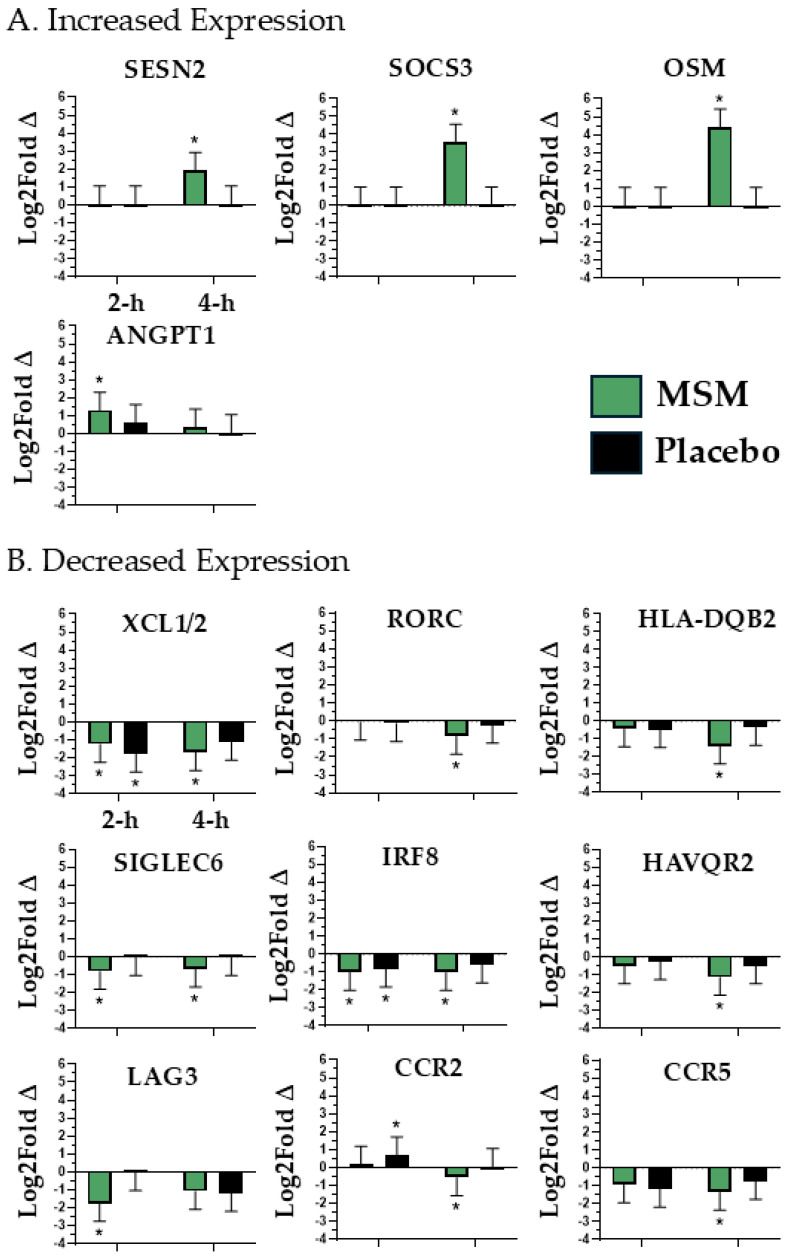
mRNAs associated with peripheral tissue inflammatory response that were either increased (**A**) or decreased (**B**) in expression with MSM following the running of a half marathon. MSM was compared to placebo. * indicates *p* < 0.05.

**Figure 4:**
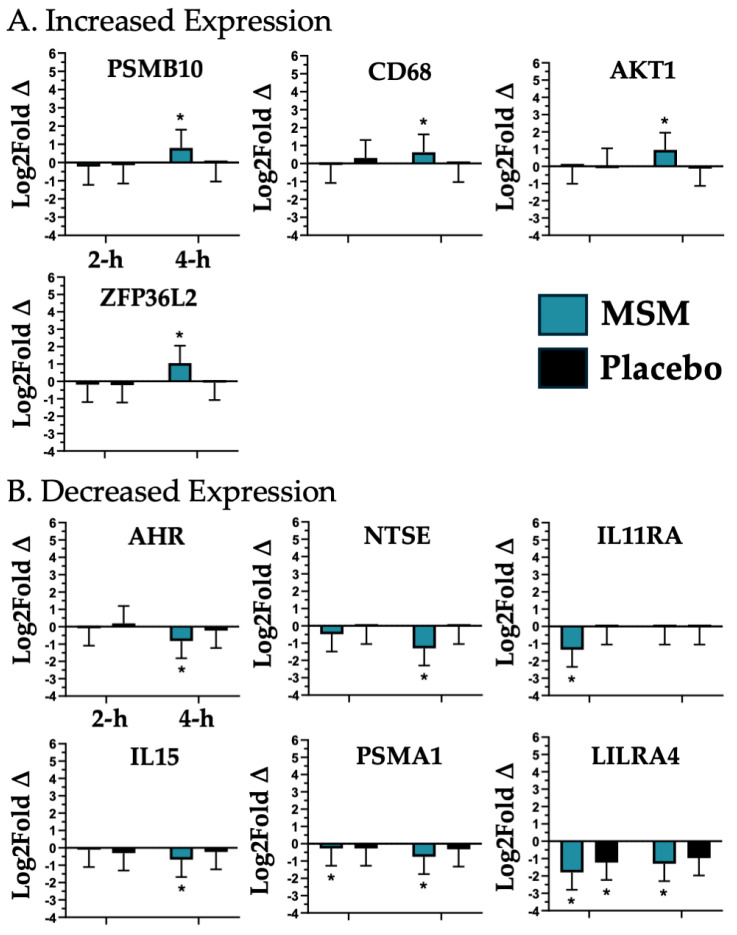
mRNAs associated with myeloid immune invasion that were either increased (**A**) or decreased (**B**) in expression with MSM following the running of a half marathon. MSM was compared to placebo. * indicates *p* < 0.05.

**Figure 5:**
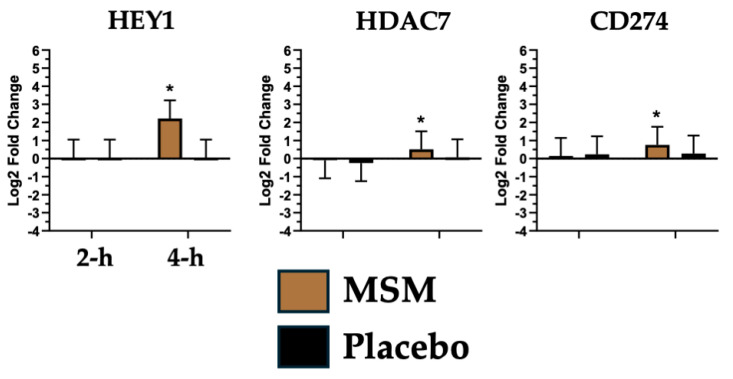
mRNA associated with notch signaling whose expression was increased with MSM following the running of a half marathon. This was compared to no change in expression with placebo. * indicates *p* < 0.05.

**Figure 6:**
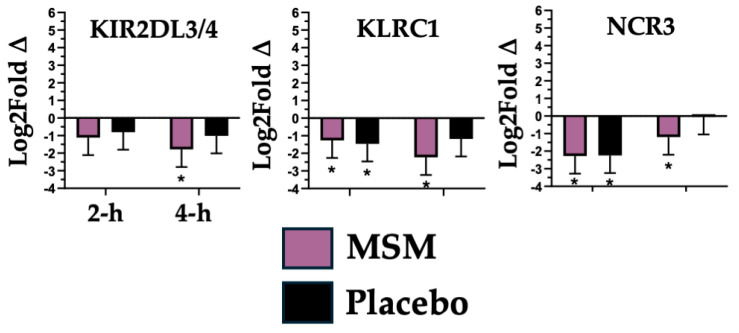
mRNA associated with natural killer (NK) cell invasion/activity whose expression was decreased by a greater degree with MSM compared to the placebo following the running of a half marathon. MSM was compared to placebo. * indicates *p* < 0.05.

Quality control measures were consistent with manufacturer recommendations and our previously published studies using Nanostring methodology [3,17,20,21,23]. Using our analysis approach and control for false discovery rate (FDR), we found 29 mRNAs whose expression was unique and statistically (*p* < 0.05) changed with MSM, but not the placebo after a half marathon race. Radar plots were plots that were generated to visualize pathway changes between placebo and MSM at 2 h (Figure 1) and 4 h (Figure 2) as identified via the Scopus AI tool; we determined that the significant mRNAs could be classified into four biological pathways for organization and discussion purposes: peripheral tissue inflammatory response (Figure 3), myeloid immune cell invasion (Figure 4), notch signaling (Figure 5), and NK cell invasion/activity (Figure 6). Figures are presented with respect to their specific sections of the discussion.

## 4. Discussion

The present study used a long-duration aerobic exercise model, which has been previously documented to result in oxidative stress and sterile inflammation [3,15,20], to evaluate the impact of MSM supplementation on the immune response to these processes. This model was used due to the well-documented rise and fall of oxidative stress and inflammation during acute exercise, and the association of these factors with chronic inflammation. When properly screened, exercise is an effective model with minimal or no adverse effects. The key finding of the present study was that 30 d of MSM supplementation significantly altered the expression of 29 mRNAs whose function was related to peripheral tissue inflammatory response, myeloid immune cell invasion, NK cell invasion/activity, and notch signaling. We identified an additional four mRNAs affected by MSM whose immediate action is not clear and may reflect novel MSM responders. The remainder of the discussion is organized to explain the mRNA changes as a function of their respective immune response pathway. The manuscript concludes with practical recommendations to provide applied applications for our observed findings.

### 4.1. Peripheral Tissue Inflammatory Response

We identified 13 mRNAs (SESN2, SOCS3, OSM, XCL1/2, RORC, HLA-DQB2, SIGLEC6, ANGPT1, HAVQR2, LAG3, CCR2, CCR5, and IRF8; see Figure 3) whose expression was altered with MSM supplementation and associated with peripheral tissue inflammatory response.

C-C motif chemokine receptor 2 (CCR2) and C-C motif chemokine receptor 5 (CCR5) play central roles in the management of oxidative stress and inflammation [24,25,26,27,28]. Specifically, CCR2 and CCR5 mediate the adhesion and subsequent transmigration of monocytes to sites of peripheral inflammation and attenuate pro-inflammatory cytokine production [24,25,26]. With respect to oxidative stress, CCR2 and CCR5 are positively correlated with increased reactive oxygen species (ROS) formation and reduced antioxidant defense [27,28]. Given their biological actions, in the context of exercise recovery, an increased CCR2 and CCR5 expression would not be beneficial as it would promote an increased state of systemic inflammation. In the present study, we found that CCR2 expression was significantly increased in the placebo at 2 h. Further, MSM was associated with a significant decrease in both CCR2 and CCR5 expression at 4 h compared to the placebo. Sialic acid-binding Ig-like lectin 6 (SIGLEC6) promotes pro-inflammatory cytokine release from mast cells [29]. Therefore, the significant reduction of this lectin with MSM at 2 h and 4 h would represent a beneficial, anti-inflammatory effect. An increased expression of Sestrin2 (SESN2) is associated with a blunting of toll-like receptor 4 response to damage-associated molecular pattern (DAMP)/pathogen-associated molecular pattern (PAMP) [30]; thus, the increased SESN2 expression observed with MSM at 4 h may represent a beneficial blunting of post-exercise inflammation. Angiopoietin 1 (ANGPT1) blocks the release of pro-inflammatory cytokines and reduces airway inflammation [31]. Therefore, the significant increase with MSM at 2 h represents a beneficial, anti-inflammatory effect. Interferon regulatory factor 8 (IRF8) is an activator of AMPK/mTOR signaling that mediates sterile inflammation and response to infection [32]. In the present study, exercise decreased IRF8 expression in both the MSM and placebo groups at 2 h; however, its expression only remained suppressed with MSM at 4 h. Given its action, this should translate to a beneficial improvement in recovery due to reduced inflammation.

Oncostatin M (OSM) facilitates the recruitment of anti-inflammatory macrophages to peripheral tissue injury sites [33]. Also, OSM knockout was associated with a significant reduction in CCR2 and CCR5 expression [33]. Like OSM, Lymphotactin (XCL1/2) is a chemokine responsible for the recruitment of natural killer and T-cells to peripheral tissue injury sites [34], and the quantity of tissue injury during exercise is positively correlated to circulating XCL1/2 concentration [35]. The overexpression of hepatitis A virus cellular receptor 2 (HAVCR2) results in a suppression of the TNF-alpha response [36], which would not be ideal for immediate exercise recovery. Our observed decrease in HAVCR2 expression at 4 h with MSM may represent an optimized TNF-alpha response to exercise, compared to the placebo. An increased expression and binding of lymphocyte activation gene-3 (LAG3) to T-cells causes cell suppression [37]. Given their respective biological actions, it would be ideal for OSM to be increased and both XCL1/2 and LAG3 to be decreased after exercise to minimize peripheral tissue inflammation. With MSM, we observed a significant increase in OSM expression and a significant decrease in XCL1/2 at both 2 h and 4 h. It is interesting to note that XCL1/2 decreased in both placebo and MSM at 2 h; however, the reduced expression was only maintained at 4 h with MSM. Suppressor of cytokine signaling 3 (SOCS3) is responsible for balancing pro-(IL-6) and anti-(IL-10) inflammatory cytokines to manage immune response [38]. Also, SOCS3 activates the NRF2 pathway, blunting ROS [39,40]. Our observed increase in SOCS3 with MSM at 4 h appears to be a beneficial adaptation, regulating post-exercise oxidative stress and inflammation.

RAR-related orphan receptor C (RORC) cell surface expression on T-cells increased in inflammatory bowel disease (IBD) [41]. Similarly, major histocompatibility complex, class II, DR alpha (HLA-DQB2) engaged in T-cell antigen presentation [42] and is found to be elevated in patients with various chronic inflammatory diseases [43]. In the present study, we observed a decreased expression of both RORC and HLA-DQB2 expression with MSM at 4 h and cautiously speculate that it may be a beneficial adaptation, although the present study may be the first to report transient changes after exercise. Collectively, the observed changes in ANGPT1, CCR2, CCR5, IRF8, OSM, SOCS3, LAG3, SIGLEC6, XCL1/2, RORC, HAVCR2, HLA-DQB2, and SESN2 expression with MSM supplementation appear to reflect a profile of an optimized peripheral inflammatory response following a half marathon race.

### 4.2. Myeloid Immune Invasion

We found that MSM was associated with a significant change in the expression of 10 mRNAs associated with myeloid immune invasion and activity (PSMB10, CD68, AKT1, ZFP36L2, AHR, NTSE, IL11RA, IL15, PSMA1, and LILRA4; see Figure 4). Aryl hydrocarbon receptor (AKT1) is required for vascular permeability and the subsequent myeloid cell invasion of injured peripheral tissue compartments [44]; thus, the increased expression observed with MSM at 4 h may represent a beneficial improvement in tissue recovery after exercise. Aryl hydrocarbon receptor (AHR) is a transcription factor reactive to environmental/metabolic stressors, resulting in increased inflammation and oxidative stress [45,46,47]. MSM significantly decreased AHR expression at 4 h, which would have a beneficial effect post-exercise. Interferon regulatory factor 8 5′-nucleotidase ecto (NT5E) encodes for CD73, whose expression marks myeloid suppressor cells [48]. Myeloid suppressor cells increase oxidative stress potential, blunting immune responsiveness [49]. In the present study, we observed a significant decrease in NT5E with MSM, which would reflect a decreased peripheral suppressor cell concentration and improved recovery compared to the placebo. Interleukin 11 receptor subunit alpha (IL11RA) ligation by IL-6-promoted myeloid suppressor cell expansion [50]; thus, the reduction we observed at 2 h with MSM would be a beneficial adaptation. Interluekin-15 (IL15) is a pro-inflammatory cytokine that also drives the movement of pro-inflammatory myeloid cells into peripheral tissue [51]. Therefore, the observed reduction of IL15 with MSM at 4 h may translate to a reduction in peripheral tissue inflammatory potential. Leukocyte immunoglobulin-like receptor A4 (LILRA4) optimizes toll-like receptor (TLR) responsiveness to DAMP/PAMP [52]. We observed that exercise caused a similar decreased LILRA4 expression at 2 h; however, the decreased expression was only maintained with MSM at 4 h, suggesting that MSM results in an extended optimization of TLR response. Proteasome 20S subunit alpha 1 (PSMA1) is positively correlated to oxidative stress, while proteasome 20S subunit beta 10 (PSMB10) plays a key role in the blunting of oxidative stress response and the activation of innate immune response [53]. We observed that MSM was associated with a decreased expression of PSMA1 and an increased expression of PSMB10 at 4 h, which given their respective actions, appears to be a beneficial effect. CD68 is a generalized marker of macrophage response [54]; thus, its increased expression with MSM at 4 h may reflect an enhanced macrophage response to muscle damage. ZFP36 ring finger protein like 2 (ZFP36L2) is capable of binding to and degrading mRNA for pro-inflammatory cytokines, thus modulating myeloid inflammatory response [55]. In the present study, we observed a significant increase in ZFP36L2 at 4 h with MSM, which may translate to a beneficial reduction in pro-inflammatory myeloid cell potential. Thus, an ideal exercise response would include decreased AHR, NT5E, PSMA1, and increased PSMB10 expression, which is the exact response we observed with MSM at 4 h. The collective changes observed in the expression of AKT1, AHR, CD68, NT5E, IL11RA, PSMA1, IL15, ZFP36L2, and PSMB10 with MSM appear to reflect a beneficial post-exercise modulation of myeloid innate invasion and pro-inflammatory potential.

### 4.3. Notch Signaling

Notch signaling plays a leading role in leukocyte communication to coordinate the peripheral immune response [56], particularly following exercise-induced tissue injury [57]. Notch regulates skeletal muscle maturation, autophagy, and recovery following injury [58,59]. We found that MSM was associated with a change in expression of three mRNAs associated with notch signaling (HEY1, HDAC7, and CD274; see Figure 5). Histone deacetylase 7 (HDAC7) allows tissue macrophages to modulate their responsiveness based on anatomical proximity to DAMP/PAMP [60]. The increased HDAC7 expression that we observed at 4 h with MSM may reflect an increased capacity for macrophages to identify DAMP/PAMP. We also observed a significant increase in Hes-related family bHLH transcription factor with YRPW motif 1 (HEY1) expression with MSM at 4 h, which mirrored the change observed for HDAC7. HEY1 is a transcription factor induced by enhanced notch signaling [56]. The CD274 molecule, also known as programmed death-ligand 1, is upregulated in response to DAMP [61], which modulates immune response. In the present study, we found that MSM was associated with increased CD274 expression at 4 h, which may represent a beneficial improvement in the typical exercise and a sterile inflammatory response to DAMP. Our observed increase in HDAC7, HEY1, and CD274 expression at 4 h with MSM may be a beneficial response to exercise, reflective of improved speed of recovery due to a coordinated immune response. A chronic elevation in notch signaling would not be ideal from a disease perspective [56]; however, given the transient nature of our observed changes following exercise, this does not reflect an increased chronic disease risk.

### 4.4. Natural Killer (NK) Cell Invasion/Activity

While not as substantial as the effects observed with generalized myeloid cell responsiveness, we also found that after exercise, MSM was associated with a reduced expression of three mRNAs (KIR2DL3/4, KLRC1, and NCR3; see Figure 5) associated with NK cell invasion and activity. Killer cell immunoglobulin-like receptor, two Ig domains and long cytoplasmic tail 3/4 (KIR2DL3/4) and killer cell lectin-like receptor C1 (KLRC1) are associated with chronic inflammatory disease onset, where their respective activation of NK cells promotes inflammation [62]. Natural cytotoxicity triggering receptor 3 (NCR3) can have both NK cell activating and inhibiting functions, depending on the context that its expression occurs [63]. In the present study, we found that MSM was associated with a significant decrease in KIR2DL3/4 (4 h), KLRC1 (2 h and 4 h, but only uniquely with MSM at 4 h), and NCR3 (2 h and 4 h, but only uniquely with MSM at 4 h). It is reasonable to speculate that MSM either reduced the systemic impact of exercise or reduced the subsequent NK cell response to it. The impact of MSM supplementation on NK cells was consistent with the observed change in myeloid cell invasion/responsiveness.

## 5. Study Limitations and Future Opportunities

The present study made use of a long-duration aerobic exercise model that is well established as a cause of transient elevations in oxidative stress and sterile inflammation [3,15,20]. Our laboratory has routinely used mRNA biomarkers to evaluate bioactive compounds in the context of exercise response, so the present findings contribute to that body of literature. Previously, the published literature described the use of MSM supplementation to mitigate the effects of various chronic disease states [9,13]. In the present study, we used long-duration aerobic exercise as a transient memetic for chronic disease. While our small sample size may be considered a limitation based on traditional outcome measures, the enrollment was consistent with other studies from our lab and others using Nanostring mRNA biomarkers. Now that we have established a profile of mRNA whose expression may be changed with MSM after exercise, we can conduct a larger study to identify additional associated mRNA and pathways that may be impacted by MSM. Our goal was to evaluate how MSM changed the post-exercise response, so we did not collect any samples prior to the pre-exercise time point. This was a practical limitation based on the recruitment/enrollment strategy used. In future studies, we need to determine if MSM also uniquely changes mRNA biomarkers at rest prior to exercise. More research is also needed to understand the long-term implications of MSM supplementation, as well as testing the identified mRNA biomarkers further in patients with chronic disease.

## 6. Conclusions

In summary, during a four-hour period following running a half marathon race, we were able to demonstrate that 30 days of prior MSM supplementation altered the expression of 29 mRNAs that may reflect an optimized immune response to oxidative stress and/or sterile inflammation. It is important to note that these effects were observed with a much lower dose of MSM than has been used in any other published exercise study. It is likely that the observed findings were found due to the precision of the Nanostring mRNA biomarker procedure used. Based on the physiologic actions of the mRNA that changed, some logical potential health outcomes of MSM (Table 1) may be that it helps with the following: (1) supports muscle recovery by improving macrophage response to exercise, (2) speeds up recovery and restoration of damaged muscle tissue, (3) supports innate immune responsiveness to DAMP, and (4) reduces and/or improves resistance to oxidative stress after exercise.

This study represents a novel expansion of previously published research by identifying mRNAs and associated pathways that may be good targets for future study. Also, future research could seek to determine if MSM also alters similar mRNAs and pathways in individuals with chronic inflammatory disease. While no study is ever perfect, the present findings represent a meaningful contribution to addressing gaps in the existing literature associated with MSM supplementation and its distinct biological actions. Despite using a much lower dose of MSM (0.5–1.0 g/d), an equally important finding was that we found MSM effects that were comparable to what others have reported with higher MSM doses (3.0 g/d) [14]. Our reported effects support the efficacy of lower doses of MSM.

## Figures and Tables

**Table 1:** Potential Health Outcomes Associated with mRNA Changes.

mRNA Biomarker	Pathway Response	Possible Health Outcome
ANGPT1, CCR2, CCR5, IRF8, OSM, SOCS3, LAG3, SIGLEC6, XCL1/2, RORC, HAVCR2, HLA-DQB2, SESN2	Peripheral Tissue Inflammatory Response	Supports Muscle Recovery Speeds Recovery of damaged Muscle Supports Innate Responsiveness to DAMP
AKT1, AHR, CD68, NT5E, IL11RA, PSMA1, IL15, ZFP36L2, and PSMB10	Myeloid Immune Invasion	Supports Muscle Recovery Speeds Recovery of damaged Muscle Reduced and/or improved oxidative stress
KIR2DL3/4, KLRC1 and NCR3	Natural Killer (NK) Cell Invasion/Activity	Supports Muscle Recovery Speeds Recovery of damaged Muscle
HDAC7, CD274 and HEY1	Notch Signaling	Supports Muscle Recovery Speeds Recovery of damaged Muscle

## Data Availability

The original contributions presented in this study are included in the article. Further inquiries can be directed to the corresponding author.

## Abbreviations

The following abbreviations are used in this manuscript:

AHR	Aryl hydrocarbon receptor;
AKT1	AKT serine/threonine kinase 1;
ANGPT1	Angiopoietin 1;
CCR2	C-C motif chemokine receptor 2;
CCR5	C-C motif chemokine receptor 5;
CD274	CD274 molecule;
CD68	CD68 molecule;
DAMP	Damage-Associated Molecular Pattern;
HAVCR2	Hepatitis A virus cellular receptor 2;
HDAC7	Histone deacetylase 7;
HEY1	Hes-related family bHLH transcription factor with YRPW motif 1;
HLA-DQB2	Major histocompatibility complex, class II, DQ beta 2;
IL11RA	Interleukin 11 receptor subunit alpha;
IL15	Interleukin 15;
IRB	Institutional Review Board;
IRF8	Interferon regulatory factor 8;
KIR2DL3/4	Killer cell immunoglobulin-like receptor, two Ig domains and long cytoplasmic tail ¾;
KLRC1	Killer cell lectin-like receptor C1;
LAG3	Lymphocyte activation gene-3;
LILRA4	Leukocyte immunoglobulin-like receptor A4;
LPS	Lipopolysaccharide;
MSM	Methylsulfonylmethane;
NCR3	Natural cytotoxicity triggering receptor 3;
NT5E	Interferon regulatory factor 8 5′-nucleotidase ecto;
OSM	Oncostatin M;
PAMP	Pathogen-Associated Molecular Pattern;
PSMA1	Proteasome 20S subunit alpha 1;
PSMB10	Proteasome 20S subunit beta 10;
RORC	RAR-related orphan receptor C;
SESN2	Sestrin 2;
SIGLEC6	Sialic acid-binding Ig-like lectin 6;
SOCS3	Suppressor of cytokine signaling 3;
XCL1/2	Lymphotactin;
ZFP36L2	ZFP36 ring finger protein like 2
